# Exercise Improves Atherosclerotic Plaque Stability Through Macrophage Autophagy and the FGF21 Signaling Pathway

**DOI:** 10.3390/ijms27114996

**Published:** 2026-05-30

**Authors:** Qingbo Li, Weidong Mao, Yao Lu, Tianrui Lu, Xiaonan Xu, Yibin Pan, Sang Ki Lee, Lifeng Wang, Ting Li, Jinming Zhou, Wei Li, Mallikarjuna Korivi

**Affiliations:** 1 College of Physical Education and Health Sciences, Zhejiang Normal University, Jinhua 321004, China; lqb1971701428@zjnu.edu.cn (Q.L.); MWD522428@zjnu.edu.cn (W.M.); gbiyyl@zjnu.edu.cn (Y.L.); ykxuxiaonan@zjnu.edu.cn (X.X.); wanglifeng@zjnu.edu.cn (L.W.); tingli@zjnu.edu.cn (T.L.); 2 Affiliated Jinhua Hospital, School of Medicine, Zhejiang University, Hangzhou 310058, China; 22318345@zju.edu.cn (T.L.); K221038@zju.edu.cn (Y.P.); 3 Department of Sport Science, College of Natural Science, Chungnam National University, Daejeon 34134, Republic of Korea; nicelsk@cnu.ac.kr; 4 Key Laboratory of the Ministry of Education for Advanced Catalysis Materials, Department of Chemistry, Zhejiang Normal University, Jinhua 321004, China; zhoujinming@zjnu.edu.cn

**Keywords:** plaque stability, atherosclerosis, treadmill running, macrophage autophagy

## Abstract

Atherosclerosis (AS) is a major driver of acute cardiovascular events, yet the mechanisms by which exercise stabilizes atherosclerotic plaque remain poorly understood. This study investigated the protective effects of a 12-week treadmill exercise training on plaque stability and macrophage autophagy in ApoE^−/−^ mice fed an atherogenic diet. Exercise significantly decreased the serum pro-inflammatory cytokine (tumor necrosis factor-α) and increased the anti-inflammatory (interleukin-10) mediator in AS mice. Histopathology analysis revealed that exercise improved plaque stability through reduced necrotic core size, increased fibrous cap thickness, and increased collagen content. These improvements were accompanied by decreased lipid accumulation, MMP-9 expression, and macrophage infiltration (CD11b) within the plaque. Mechanistically, exercise activated plaque autophagy, evidenced by increased LC3B fluorescence, elevated LC3II/I ratio, restoration of Beclin-1, and degradation of p62. Notably, exercise-induced autophagy is specific to plaque-resident macrophages, as demonstrated by strong colocalization of LC3B and CD11b fluorescent signals (Pearson’s correlation coefficient = 0.56). Furthermore, exercise restored fibroblast growth factor 21 (FGF21) levels in both circulation and plaque while concurrently suppressing downstream PI3K/Akt/mTOR signaling. Collectively, these findings demonstrated that exercise promotes plaque stability by reducing lipid accumulation, macrophage infiltration, MMP-9 expression, and activation of FGF21. This protection is likely mediated by the activation of macrophage autophagy, specific to plaque-resident macrophages, indicating the cardioprotective benefits of aerobic exercise against AS.

## 1. Introduction

Atherosclerosis (AS) is a chronic cardiovascular disease (CVD) characterized by the formation of lipid plaques within the arterial wall [1]. During the progression of AS, the continuous accumulation of lipids and inflammatory cytokines within the plaque results in an increased size and compromised structural integrity, leading to instability and vulnerability to rupture [2]. Plaque rupture is a critical clinical event that causes acute complications, such as myocardial infarction (MI) and cerebral thrombosis [2]. Plaque size, collagen content, fibrous cap thickness, and lipid content are crucial in plaque stability [3]. Recent studies have emphasized the importance of therapeutic strategies in stabilizing the vulnerability of plaque and decreasing the incidence of cardiovascular events [4,5,6].

Atherosclerotic plaque stability is typically maintained by two cellular events, namely autophagy and apoptosis [7]. Autophagy is a dynamic degradation phenomenon, regulated by key autophagic proteins, LC3 II/I, Beclin 1, and p62, which can mediate either survival or detrimental effects [8,9]. Autophagy within the AS plaque is orchestrated through an integrated signaling network across major constituent cell types, including endothelial cells, vascular smooth muscle cells (VSMCs), and macrophages [10,11]. Notably, specialized forms, like lipid autophagy and macrophage autophagy, occur in different stages of AS, where they function to prevent plaque rupture or promote stability [9,12]. For instance, macrophage autophagy has been shown to strengthen the fibrous cap and increase plaque stability by suppressing the secretion of matrix metalloproteinases (MMPs) [13]. Pharmacological modulation of this pathway has demonstrated therapeutic potential in treating AS. The anti-inflammatory compound, like quercetin, has been shown to ameliorate AS by increasing the LC3 II/I ratio to promote macrophage autophagic activity in mice fed a high-fat diet (HFD) [14]. Mechanistically, increased macrophage autophagy can be achieved by activating direct phosphorylation of serine/threonine kinase (Akt) through phosphatidylinositol 3-kinase (PI3K) [15], and this axis is vital for maintaining plaque stability [16]. Furthermore, overexpression of fibroblast growth factor 21 (FGF21) inhibits the downstream PI3K/Akt/mTOR signaling pathway and promotes autophagic responses [17]. Consistent with this, elevated FGF21 promotes autophagy, whereas knockdown of FGF21 resulted in a significant decrease in autophagy in the aorta [18].

Aerobic exercise is an effective intervention to mitigate the formation and progression of atherosclerotic plaques [19]. A previous study demonstrated that swimming exercise significantly reduces plaque area, concurrently lowering serum levels of MMP-9 and inflammatory cytokines, interleukin-6 (IL-6), and IL-1β [20]. Voluntary exercise is also reported to reduce atherosclerotic plaque formation in ApoE^−/−^ mice, possibly by stimulating endothelial cell autophagy via improved IL-1 signaling [21]. Exercise training inhibits plaque formation and promotes serum β-hydroxybutyric acid (BHB) in ApoE^−/−^ mice, and treatment of peripheral macrophages with BHB activated autophagy [22]. Anti-atherosclerotic effects of exercise appear to be associated with enhanced autophagy in the aortic sinus, yet the detailed molecular mechanisms remain unexplored. Kwon et al. observed increased expression of autophagy markers (LC3II, ATG7) and decreased p62 in the liver of exercised mice [23]. Furthermore, exercise training is reported to upregulate FGF21 expression in muscle and adipose tissues of obese mice [24].

The available evidence shows that exercise can promote plaque stability by endothelial cell or peripheral macrophage autophagy [21,22]. However, whether exercise can directly promote macrophage accumulation or macrophage autophagy within plaque-resident macrophages remains unknown. In addition, the role of FGF21 in mediating the effects of exercise on AS plaque stability has not yet been verified. Therefore, we aimed to investigate the effect of aerobic exercise on atherosclerotic plaque stability and autophagy in the ApoE^−/−^ mouse model of AS. We further explored whether exercise-induced effects are specific to plaque-resident macrophages, and examined the role of FGF21 and its downstream signaling proteins in regulating autophagic activity.

## 2. Results

In this study, we examined the anti-atherosclerotic effects of exercise training in the AS mouse model (ApoE^−/−^ mice fed an atherogenic diet). Body weight, inflammatory markers, and plaque stability were assessed in wild-type (control and exercise [Ex]) and ApoE^−/−^ mice (AS and AS plus Ex). Additionally, biomarkers of autophagy, macrophage-specific autophagy, and FGF21 signaling were evaluated within the plaque, and the results were compared between AS and AS + Ex groups.

### 2.1. Exercise Ameliorates the Inflammatory Response in Atherosclerotic Mice

**Figure 1:**
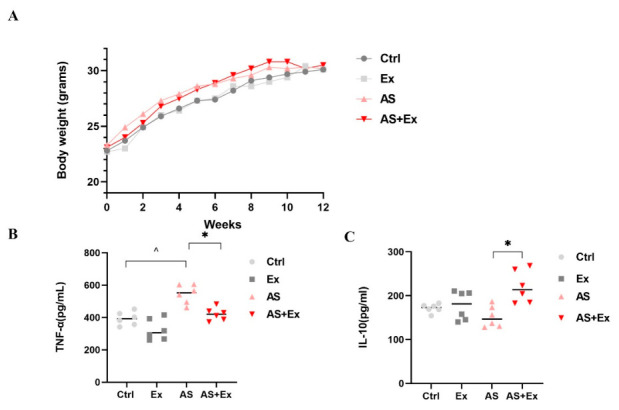
(**A**) Changes in body weight of mice in control (Ctrl), exercise (Ex), atherosclerosis (AS), and atherosclerosis plus exercise (AS + Ex) groups. (**B**) Concentrations of serum tumor necrosis factor-alpha (TNF-α), and (**C**) interleukin-10 (IL-10). Data are presented as mean ± SEM (n = 6). Significant differences: ^ *p* < 0.05 (Ctrl vs. AS), * *p* < 0.05 (AS vs. AS + Ex).

As shown in Figure 1, no significant differences in body weight were observed among the groups during and at the end of treatment (Figure 1A). To determine the effects of exercise training on inflammation, serum pro-inflammatory and anti-inflammatory cytokines were measured. The AS group displayed significantly increased TNF-α levels compared to the control group (*p* < 0.05), while the AS + Ex group showed a significantly decreased TNF-α concentration compared to the AS. Furthermore, exercise was associated with a significant increase in IL-10 levels compared to the AS group (*p* < 0.05) (Figure 1B,C). These results suggest that aerobic exercise is beneficial in inflammatory homeostasis in the AS model.

### 2.2. Exercise Promotes Plaque Stability

**Figure 2:**
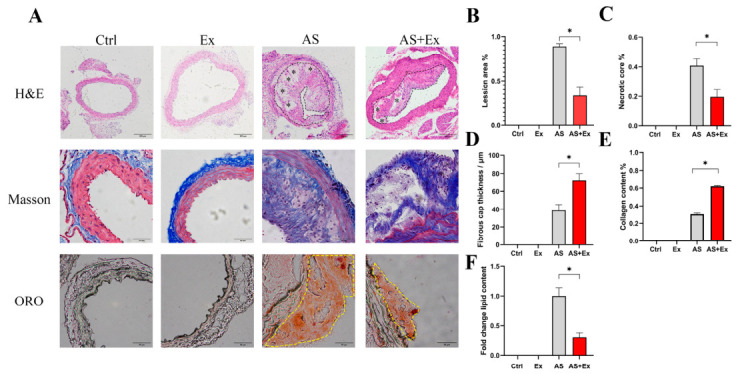
Effect of exercise on atherosclerotic plaque stability in atherosclerosis (AS) and atherosclerosis plus exercise (AS + Ex) groups. (**A**) Representative images of H & E staining showing lesion area, necrotic core, and fibrous cap thickness (upper panel, scale bar = 200 µm; * means necrotic core in AS and AS + Ex). Representative images of Masson’s staining showing the collagen fibers (middle panel) and oil red O staining showing the lipid content (lower panel) in aortic plaques (scale bar = 50 µm). (**B**) Quantitative data of aortic plaque area, (**C**) necrotic core size, (**D**) fibrous cap thickness, (**E**) collagen fiber content, and (**F**) lipid content within plaques. Histograms represent quantified data from four images per group, and are presented as mean ± SEM (n = 4). * *p* < 0.05 indicates a significant difference between AS and AS + Ex groups.

The effect of aerobic exercise on atherosclerotic plaque stability was evaluated using histopathological analysis, and the results are presented in Figure 2. Histopathological images of the control and exercise groups demonstrated normal morphology in the thoracic aorta. In contrast, the AS group exhibited substantial plaque deposition, occupying nearly the entire vascular lumen. The AS group also exhibited increased necrotic core formation and reduced fibrous cap thickness (Figure 2A, upper panel, B–D). It is worth noting that exercise training in the AS + Ex group attenuated plaque expansion and size by decreasing the necrotic core and concurrently thickening the fibrous cap (Figure 2A–D).

Results from the Masson staining images revealed a significant increase (*p* < 0.05) of plaque collagen content in the AS + Ex group compared to the AS group (Figure 2A, middle panel, E). Microphotographs from the oil red O (ORO) assay demonstrated pronounced lipid accumulation in the subintimal layer of aortic plaques within the AS group. However, this lipid accumulation appears to be significantly reduced (*p* < 0.05) with exercise (Figure 2A bottom panel, F). These findings suggest that exercise training can promote plaque stability by reducing plaque area, thickening the fibrous cap, and inhibiting lipid deposition, while restoring collagen content.

### 2.3. Exercise Reduces MMP-9 and CD11b in Atherosclerotic Plaques

**Figure 3:**
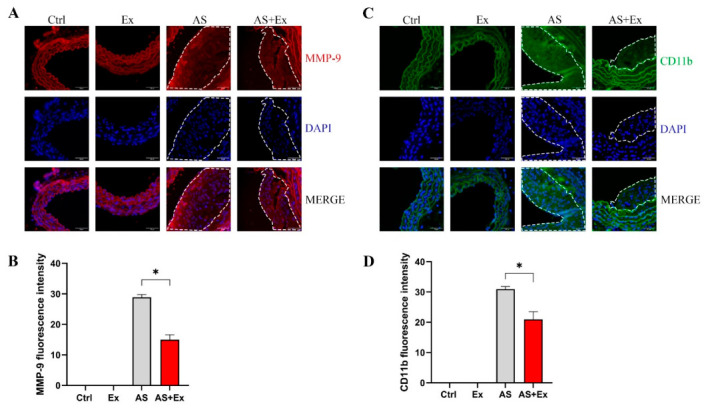
Effect of exercise on MMP-9 and macrophage infiltration (CD11b) in the plaque of atherosclerosis (AS) and atherosclerosis plus exercise (AS + Ex) groups. (**A**) Representative immunofluorescence images of MMP-9. Scale bar: 50 µm. Red: MMP-9; blue: DAPI-stained nuclei. (**B**) Quantification of MMP-9 fluorescence intensity presented as histograms (n = 4). (**C**) Representative immunofluorescence images of CD11b. Scale bar: 50 µm. Green: CD11b; blue: DAPI-stained nuclei. (**D**) Quantification of CD11b fluorescence intensity presented as histograms (n = 4). Data are presented as mean ± SEM. Significant differences (* *p* < 0.05) between AS and AS + Ex groups.

Immunofluorescence staining was performed to evaluate the effects of exercise on MMP-9 and CD11b in plaques. Images revealed that the fluorescence intensities of MMP-9 (Figure 3A,B) and CD11b (Figure 3C,D) were lower in the AS + Ex group compared to the AS. The quantified fluorescence intensities showed a 40.6% decrease in MMP-9 (*p* < 0.05, Figure 3B) and a 31.2% decrease in the CD11b-positive area (*p* < 0.05, Figure 3D) within plaques of the AS + Ex group relative to the AS group. These data imply that exercise suppresses macrophage-derived MMP-9 and macrophage infiltration in atherosclerotic plaques.

### 2.4. Exercise Enhances Autophagic Activity in Atherosclerotic Plaques

**Figure 4:**
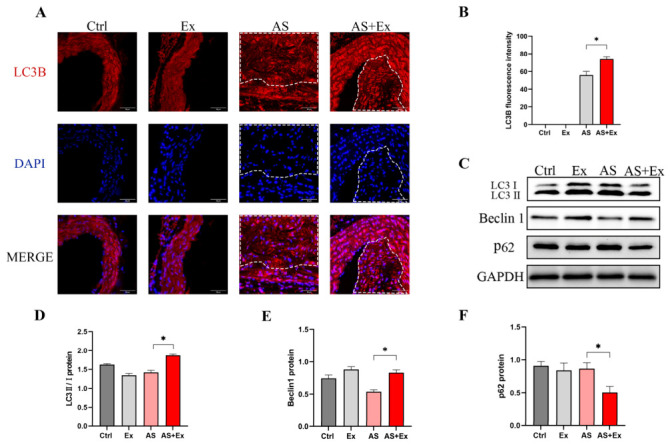
Effect of exercise on autophagy markers within the plaque of atherosclerosis (AS) and atherosclerosis plus exercise (AS + Ex) groups. (**A**) Representative immunofluorescence images of LC3B (scale bar: 50 µm, red: LC3B, blue: DAPI-stained nuclei). (**B**) Quantification of LC3B fluorescence intensity presented as histograms (n = 4). (**C**) Representative Western blot images of LC31/II, Beclin 1, and p62 proteins. GAPDH was used as an internal control. Quantified protein intensities of LC31/II (**D**), Beclin 1 (**E**), and p62 (**F**) are shown as representative histograms (4 assessments per group). Data presented as mean ± SEM. Significant differences (* *p* < 0.05) between AS and AS + Ex groups.

To study whether exercise promotes autophagy in the plaque, autophagic markers were estimated using immunofluorescence and Western blot analyses. Results from the immunofluorescence images showed elevated red-stained intensity in the AS + Ex group, indicating increased LC3B expression; however, such accumulation was not noticed in the AS group (Figure 4A). Quantitative analysis demonstrated a 30.3% higher LC3B expression in the AS + Ex group compared to the AS group (*p* < 0.05, Figure 4B). Subsequent Western blot results also demonstrated the increased LC3II/I protein in the AS + Ex group and that the quantified LC3II/I was significantly higher in the AS + Ex group compared to AS (Figure 4C,D). Furthermore, Beclin 1 protein was lower, and p62 was stable in the AS group, indicating impaired autophagic flux. Nevertheless, exercise training appears to restore Beclin 1 and suppress p62 levels in the plaque (Figure 4C–F). Taken together, these findings suggest that exercise training promotes autophagic activity in the atherosclerotic plaques.

### 2.5. Exercise-Induced Autophagy Specific to Macrophages in Plaque

**Figure 5:**
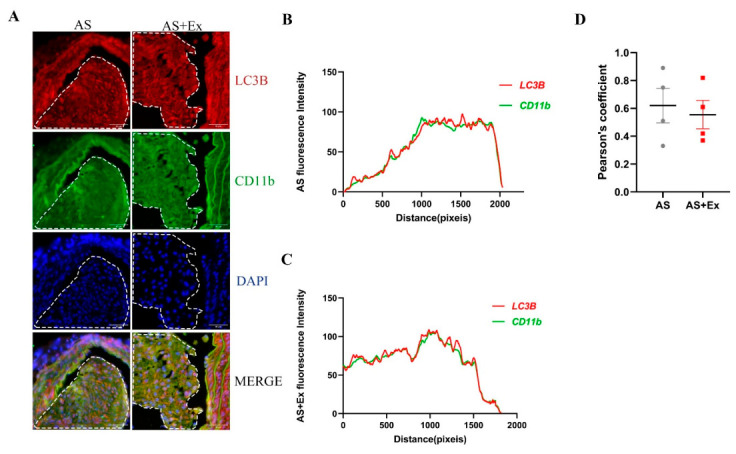
Colocalization of autophagic marker LC3B and macrophage marker CD11b in the plaque of atherosclerosis (AS) and atherosclerosis plus exercise (AS + Ex) groups. (**A**) Representative images showing LC3B (red) and CD11b (green) colocalization in the AS (left panel) and AS + Ex (right panel) groups. Nuclei were counterstained with DAPI (blue). Scale bars: 50 µm. (**B**,**C**) Intensity profiles of target biomarkers generated via ImageJ 1.54d line-scan analysis. (**D**) Pearson’s correlation coefficient (PCC) was calculated for AS and AS + Ex groups, and the PCC value > 0.5 means high colocalization in plaque.

To determine whether exercise promotes autophagy in plaque-resident macrophages, we assessed the colocalization of the autophagic marker (LC3B) and macrophage marker (CD11b) using a confocal microscope. We found positive fluorescent signals for both LC3B and CD11b in the plaques of AS and AS + Ex groups, indicating the presence of autophagy within the plaques (Figure 5A).

We then merged both LC3B and CD11b intensity signals and plotted their spatial distribution (distance pixels) to determine the association between these markers (Figure 5B,C). The intensity trajectories for LC3B and CD11b were highly overlapped, indicating tight colocalization within the plaque. A strong correlation between LC3B and CD11b was observed in both the AS and AS + Ex groups, which was further confirmed by Pearson’s correlation analysis (AS: r = 0.62 ± 0.11; AS + Ex: r = 0.56 ± 0.09) (Figure 5D). Collectively, these findings suggest that exercise-induced autophagy may occur specifically within plaque-resident macrophages.

### 2.6. Exercise Modulates FGF21 Signaling Pathway in Plaques

**Figure 6:**
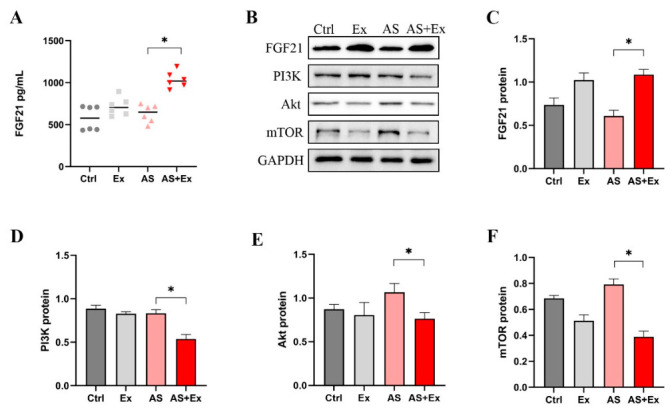
Effect of exercise on FGF21 and its downstream protein in the plaque of atherosclerosis (AS) and atherosclerosis plus exercise (AS + Ex) groups. (**A**) Exercise restored serum FGF21 concentrations (n = 6). (**B**) Representative Western blot images of FGF21, PI3K, Akt, and mTOR proteins; GAPDH was used as an internal control. Quantified protein concentrations FGF21 (**C**), PI3K (**D**), Akt (**E**), and mTOR (**F**) were presented as histograms (n = 4). Data are presented as mean ± SEM. Data presented as mean ± SEM (n = 4). Significant differences: * *p* < 0.05 (AS vs. AS + Ex).

We measured FGF21 concentrations in serum and FGF21 protein levels in aortic plaque. We found significantly increased circulating FGF21 in the AS + Ex group compared with the AS group (*p* < 0.05, Figure 6A). Similarly, the FGF21 protein levels within the plaque were also significantly elevated in the AS + Ex group compared with the AS (*p* < 0.05, Figure 6B,C). We then examined the key proteins involved in the FGF21 signaling pathway. Western blot results (Figure 6B) revealed that PI3K, Akt, and mTOR protein levels were significantly reduced in the AS + Ex group compared with the AS (*p* < 0.05, Figure 6D–F). These results indicate that exercise restores plaque FGF21 protein while downregulating the PI3K/Akt/mTOR axis, suggesting a potential mechanism for autophagy modulation.

## 3. Discussion

Our findings demonstrate that aerobic exercise training stabilizes the atherosclerotic plaque in ApoE^−/−^ mice, potentially by decreasing plaque area, increasing fibrous cap thickness, diminishing necrotic lipid cores, and restoring collagen content. These structural improvements with exercise were accompanied by a reduction in plaque macrophages and macrophage-derived MMP-9, suggesting macrophage modulation in exercise-induced stabilization. Mechanically, exercise enhances autophagy within plaques, as indicated by the restoration of LC3II/I and Beclin 1 expression, alongside a reduction in p62 protein levels. This autophagic response appears to be localized predominantly to plaque-resident macrophages, as evidenced by the spatial overlap of LC3B and CD11b fluorescent signals (Pearson’s correlation: r = 0.56). These findings suggest that exercise may selectively induce autophagic activity in plaque macrophages. Next, we identified FGF21 as a potential upstream regulator of this process. Exercise restored plaque FGF21 expression in the AS + Ex group, while suppressing the PI3K/Akt/mTOR pathway, which is a canonical inhibitory axis of autophagy. Taken together, these findings suggest that exercise training may stabilize atherosclerotic plaques by activating macrophage autophagy, potentially via FGF21-mediated suppression of PI3K/Akt/mTOR signaling pathways.

The structural integrity of atherosclerotic plaques is a critical determinant of clinical outcomes, with vulnerable lesions characterized by thin fibrous caps, large necrotic lipid cores, and reduced collagen content [2,25]. Therefore, identifying the most vulnerable plaques enables the identification of high-risk lesions and allows the identification of possible preventive measures before acute coronary events [26]. In this study, exercise training promoted plaque stability in the AS model (ApoE^−/−^ mice), which is comparable to the effect of statin therapy [27]. The improved plaque stability with exercise may be attributed to increased fibrous cap thickness and collagen deposition, while decreasing necrotic core formation and subintimal lipid accumulation. These structural modifications collectively reduce plaque vulnerability and promote plaque stability [28]. The increased collagen content by exercise is likely to counteract the mechanical weakness that predisposes plaques to rupture [29], whereas the reduction in intraplaque lipids may reflect exercise-induced improvements in systemic cholesterol efflux and metabolic homeostasis [22]. Inflammatory cytokines that negatively impact plaque stability have been shown to improve with exercise in an atherosclerosis mouse model, which may promote plaque stability [30]. In our study, exercise decreased the pro-inflammatory cytokine (TNF-α) and restored the anti-inflammatory cytokine (IL-10) in ApoE^−/−^ mice. Collectively, these effects of exercise on plaque architecture, lipid metabolism, and inflammation likely converge to promote overall plaque stability.

Macrophages play a central role in atherosclerotic plaque instability, largely through the secretion of MMPs, particularly MMP-9 [31], which degrades collagen within the fibrous cap and predisposes plaques to rupture [32,33]. Elevated MMP-9 is also associated with greater macrophage infiltration into lesions, reduced collagen, and increased elastin fiber fragmentation [32,34]. In our study, exercise significantly reduces both CD11b-positive macrophage infiltration and MMP-9 expression in aortic plaque, consistent with previous reports that exercise training downregulates MMP-9 expression, macrophage infiltration, and reduces plaque progression [35,36]. A greater decrease in macrophages and MMP-9 provides a mechanistic link between exercise-induced modulation of inflammatory cell content and preservation of the structural integrity of the plaque. This interpretation is supported by preclinical evidence showing that MMP-9 ablation attenuates lesion development in ApoE^−/−^ mice [37] and that MMP-9 promotes monocyte recruitment into atherosclerotic lesions [38]. Since macrophages are the key players in plaque stability or rupture, a decrease in macrophage number by exercise may reasonably explain the improved plaque stability [35]. Collectively, these results suggest that exercise promotes plaque stability, at least in part by mitigating macrophage-mediated proteolytic activity within the plaque environment.

One of the key findings in our study is that exercise training activated autophagy in plaques of ApoE^−/−^ mice, as evidenced by increased LC3 II/I and Beclin 1 proteins alongside reduced p62 levels. Autophagic response in atherosclerosis exerts protective effects (decreased risk of plaque rupture) by preventing leucocyte aggregation and monocyte infiltration and alleviating inflammatory response within the plaque [10,39]. Clinical data showed that impaired autophagy (decreased LC3 and Beclin 1) increased plaque vulnerability in patients with acute coronary syndrome [40]. Meanwhile, decreased autophagic proteins (LC3 and Beclin 1) in the aorta by exercise are associated with alleviation of atherosclerotic plaque burden in ApoE^−/−^ mice [20]. Okutsu et al. demonstrated that aerobic exercise upregulates autophagic protein (LC3 II/I) in aortic endothelial cells and stimulates autophagy in ApoE^−/−^ mice [21]. Their in vitro findings further indicated that endothelial autophagy via improved IL-1 signaling is probably involved in protection against atherosclerosis [21]. Another study has shown that exercise promotes plaque stability by increasing serum BHB in ApoE^−/−^ mice, and BHB treatment to peripheral macrophages increased autophagy (increased LC3II and decreased p62) [22]. These findings suggest that autophagy activation is one of the mechanisms through which exercise promotes plaque stability; however, the specific cell type involved in autophagy within plaque remained unclear.

For the first time, here we demonstrated that exercise-induced autophagy in atherosclerotic plaques occurs predominantly in plaque-resident macrophages. This phenomenon was evidenced by the spatial overlap of both LC3B and CD11b fluorescent signals, with tightly correlated intensity trajectories in the plaque of exercise mice. Autophagy of vascular endothelial and VSMCs can promote plaque stability [41]; however, macrophage autophagy is particularly relevant to plaque stability, as these cell types are intrinsically involved in inflammation- and MMP-mediated matrix degradation [12]. Particularly, macrophage autophagy in the early stage of atherosclerosis is associated with decreased accumulation of foam cells (inhibiting plaque formation), while macrophage autophagy in the late stage is associated with decreased inflammation in plaque and improved plaque stability [9]. Furthermore, clearance of macrophages via autophagy is also associated with a reduced plaque burden, decreased vulnerability index, and lower incidence of plaque rupture [16]. Therefore, exercise-induced macrophage autophagy represents a plausible mechanism underlying the observed improvements in plaque stability alongside reduced inflammatory mediators and MMP-9 activation.

We next sought to identify upstream regulators of exercise-induced autophagy in the plaques. We found that exercise training significantly increased both circulating and plaque FGF21 levels in ApoE^−/−^ mice, accompanied by suppression of the autophagy inhibitory axis, the PI3K/Akt/mTOR pathway, within the plaques. This relationship aligns with the existing evidence that FGF21 promotes autophagy by inhibiting the PI3K/Akt/mTOR signaling pathway in prostate cancer cells [17]. Furthermore, FGF21 deficiency in the aorta is associated with decreased autophagic proteins, increased lipid accumulation, and exacerbation of atherosclerosis, whereas FGF21 activation reverses these pathological features in ApoE^−/−^ mice [18]. In our study, increased FGF21 appears to be correlated with increased plaque LC3B and Beclin 1 proteins and decreased p62 levels. We speculate that exercise-induced autophagy may be associated with FGF21-mediated suppression of the PI3K/Akt/mTOR pathway in the plaque. This mechanistic link is partially supported by a previous report showing increased autophagy while inhibiting PI3K/Akt/mTOR signaling in the hippocampus of diabetic mice after high-intensity interval training [42]. Our findings suggest that aerobic exercise could stabilize atherosclerotic plaques by promoting macrophage autophagy, possibly via the FGF21/PI3K/Akt/mTOR signaling axis. Nevertheless, due to a lack of strong evidence, our findings cannot establish that FGF21 mediates the anti-atherosclerotic effects of exercise in the ApoE^−/−^ mice. Further studies with the FGF21 knockout model are necessary to demonstrate the association between FGF21 signaling and the anti-atherosclerotic effects of exercise.

## 4. Limitations

Despite the beneficial effects of exercise against atherosclerosis, our study has a few limitations. First, no pharmacological inhibitors were used to verify the exercise-induced autophagy within plaque cells. Second, the FGF21 knockout model was not used in our study to demonstrate the association between exercise-induced autophagy and FGF21-mediated signaling. We reported that macrophage autophagy within the plaque promotes plaque stability, yet the role of VSMCs in plaque stability cannot be ruled out in the AS model.

## 5. Materials and Methods

### 5.1. Animal Care and Grouping

A total of 24 male mice comprising ApoE^−/−^ (n = 12) and C57BL/6J wild-type littermates (n = 12), aged 6 weeks (body weight 20 ± 1 g), were procured from the Beijing Vital River (Jiaxing, China). Both types were maintained under standardized laboratory conditions in the animal center at Zhejiang Normal University. The animal facility is with controlled environmental parameters: ambient temperature 23 ± 2 °C, humidity 50–60%, and a 12:12 h light–dark cycle. The food and water were ad libitum to all animals. Following a 7-day acclimatization period, the C57BL/6J wild-type mice were categorized into control (Ctrl) and exercise (Ex) groups, while the ApoE^−/−^ mice were categorized into atherosclerosis (AS) and atherosclerosis plus exercise (AS + Ex) groups. Mice in control and exercise groups received a standard laboratory diet (fat 5.0%, Research Diets, New Brunswick, NJ, USA). ApoE^−/−^ mice in the AS and AS + Ex groups were fed an atherogenic diet (fat 42.0%, Research Diets, New Brunswick, NJ, USA) for 12 weeks. Food intake was recorded daily, and changes in body weights were recorded weekly for all groups.

### 5.2. Aerobic Exercise

Before performing the final exercise protocol, mice in exercise groups were familiarized and trained on a treadmill (ZH-PT model, Anhui Zhenghua Biologic Apparatus Facilities, Huaibei, China). The starting running speed on day 1 was 10 m/min for 10 min. From day 2, both speed and duration of exercise were progressively increased by 1 m/min and 10 min per day until reaching the target speed of 15 m/min for 60 min [22]. The final exercise training protocol consisted of a 15 m/min running speed for 60 min with 2 min rest intervals every 15 min, on a 5-degree inclined treadmill. Training sessions were scheduled from Monday to Friday between 5:00 and 7:00 PM, for 12 weeks. All animals in the exercise groups completed the whole training protocol.

### 5.3. Blood and Aorta Tissue Collection

Twenty-four hours after the final exercise session with 12 h fasting, mice were anesthetized with an intraperitoneal injection of urethane (2 g/kg body weight). Upon confirming deep anesthesia by the absence of corneal and pain reflexes, euthanasia was performed by cervical dislocation. Under 12 h fasting, approximately 0.6 to 0.7 mL of blood sample was collected from the inferior vena cava into an anticoagulant tube, using a heparinized syringe. The blood samples were centrifuged at 3500 rpm for 15 min, and serum was collected. Next, the thoracic aorta was surgically excised using a scalpel and immediately placed in phosphate-buffered saline (PBS). A portion of the aorta was used for histopathological studies, and the remaining tissue was frozen at −80 °C for additional biochemical assays.

### 5.4. Assessment of Inflammatory Cytokines and FGF21

The changes in pro-inflammatory cytokine tumor necrosis factor-alpha (TNF-α) and anti-inflammatory cytokine interleukin-10 (IL-10) were determined in the blood using ELISA kits. The commercially available ELISA kits were obtained from Ruixin Biotech (Quanzhou, China). The changes in circulatory concentrations of FGF21 were estimated according to the instructions described in the ELISA kit (Ruixin Biotech, Quanzhou, China). The optical densities of TNF-α, IL-10, and FGF21 were read on a UV–visible spectrophotometer (Thermos Scientific, Waltham, MA, USA).

### 5.5. Histopathological Studies

#### 5.5.1. Assessment of Atherosclerotic Plaque Stability

The aorta was fixed in 4% paraformaldehyde (pH 7.4) for 24 h at 4 °C. The tissues were processed sequentially with ethanol dehydration (50–100% gradient) and embedded in paraffin wax. To preserve anatomical integrity, the embedded blocks were either cryopreserved at −20 °C or sectioned immediately (5 μm thickness) using a rotary microtome (Leica RM2235, Wetzlar, Germany). Hematoxylin and eosin (H & E) staining was used to examine plaque morphology and quantify lesion area. The plaque area percentage was calculated as the ratio of the plaque area to the total intimal area × 100%. The boundary of the necrotic core within the plaque was traced, and the percentage of the necrotic core area relative to the total plaque area was calculated. Five random points were selected along the fibrous cap, and the vertical distance from the luminal surface of the cap to the top of the necrotic core was measured at each point. The calculated average value of five measurements of fibrous cap thickness was presented as micrometers (μm). The microphotographs were observed under the fluorescence microscope (Leica, Weztlar, Germany). The intensities of the lesion area, necrotic core cells, and fibrous cap thickness were quantified using ImageJ software 1.54d (National Institutes of Health, Bethesda, MD, USA).

#### 5.5.2. Determination of Collagen Content and Lipid Accumulation

The collagen content within plaques was assessed via Masson’s trichrome staining, with aniline blue counterstaining differentiating collagen fibers from the smooth muscle components. Fresh aorta was embedded in optimal cutting temperature (OCT) compound, and then carefully sectioned into 10 μm thick slices using a cryostat (Leica CM1860). The freshly sliced sections were mounted on glass slides. The quantified collagen content was presented as a percentage. Next, oil red O (ORO) staining was performed to determine the lipid content within the plaques, and data were presented as fold change. Computational analysis for collagen content and lipid deposition was performed using ImageJ software 1.54d.

### 5.6. Immunofluorescence Staining

Tissue sections of the aorta were prepared and incubated with a primary antibody mixture of LC3B (dilution, 1:200), CD11b (dilution, 1:50), and MMP-9 (dilution, 1:100) at 4 °C overnight. All three antibodies were obtained from Abclonal, Wuhan, China. The sections were protected from light exposure, and Goat pAb to Rb IgG (dilution, 1:200) and Goat pAB to Ms IgG (dilution, 1:200) secondary antibody mixture was added. The secondary antibodies were purchased from Abcam, Cambridge, UK. Then, the microscope slides were incubated for 1 h at room temperature before sealing with a DAPI-containing sealer (Beyotime, Shanghai, China). Finally, the stained slides were examined under a fluorescence microscope (Leica, Wetzlar, Germany). The fluorescence intensity for all biomarkers was quantified using ImageJ software 1.54d.

### 5.7. Western Blot

Total protein extraction from the aorta was performed using radioimmunoprecipitation assay (RIPA) lysis buffer supplemented with protease/phosphatase inhibitor cocktail (Thermo Fisher Scientific, Waltham, MA, USA). Protein levels were quantified using the Rapid Gold Bicinchoninic Acid (BCA) assay kit (Thermo Fisher Scientific, Waltham, MA, USA). A measurement of 30 µg of protein aliquots was separated using 8–15% sodium dodecyl sulfate (SDS)–polyacrylamide gels. Next, the separated proteins were transferred onto polyvinylidene fluoride (PVDF) membranes (Bio-Rad, CA, USA) followed by blocking with 5% skimmed milk (Bio-Rad, CA, USA) for one hour. The membranes were then washed thrice with Tris-buffered saline containing 0.5% Tween^®^20 detergent (TBST), and incubated overnight with primary antibodies, such as Beclin 1 (1:1000, 11306–1-AP, Proteintech, Wuhan, China), PI3K (1:1000, 20584–1-AP, Proteintech, Wuhan, China), Akt (1:2000, 10176–2-AP, Proteintech, Wuhan, China), mTOR (1:5000, 66888–1-Ig, Proteintech, Wuhan, China), GAPDH (1:100,000, 60004–1-Ig, Proteintech, Wuhan, China), LC3B (1:1000, A19665, Abclonal, Wuhan, China), FGF21 (1:1000, A23463, Abclonal, Wuhan, China), and p62 (1:20,000, Abclonal, Wuhan, China). Secondary antibody incubation (goat anti-mouse, Proteintech; goat anti-rabbit, Cell Signaling Technology) proceeded for 2 h at room temperature. After final TBST washes, chemiluminescent detection was employed using enhanced chemiluminescence (ECL) reagent (Thermo Fisher Scientific, Waltham, MA, USA) and a Bio-Rad imaging system (Hercules, CA, USA). ImageJ software 1.54d was used to quantify the protein band intensities.

### 5.8. Statistical Analysis

All data are presented as mean ± standard error of the mean (SEM). Descriptive statistics were calculated using SPSS software version 27.0 (IBM, Chicago, IL, USA). Independent sample *t*-tests (for histopathological slice analysis) and one-way analysis of variance (ANOVA) (for the remaining data) were employed to determine the statistical significance, followed by Bonferroni post hoc tests. A threshold of *p* < 0.05 was considered statistically significant. The degree of fluorescent colocalization (for LC3B and CD11b in plaque) was quantified by calculating the Pearson’s correlation coefficient (PCC), with values ranging from 0 (no colocalization) to one (complete colocalization).

## 6. Conclusions

Our findings demonstrate that aerobic exercise training promotes atherosclerotic plaque stability in ApoE^−/−^ mice, which is attributed at least in part to the activation of autophagy in plaque-resident macrophages. This macrophage-specific autophagy was evidenced by colocalization of CD11b and LC3B within plaques, alongside increased LC3B II/I and Beclin 1 levels and reduced p62. Exercise training appears to restore FGF21 while suppressing the PI3K/Akt/mTOR signaling pathway, suggesting that this axis acts as an upstream regulator of exercise-induced autophagy. Our findings indicate that aerobic exercise training stabilizes atherosclerotic plaques, possibly by promoting plaque-resident macrophage autophagy, highlighting the anti-atherosclerotic effects of exercise.

## Data Availability

The original data presented in this study are included in the article. Further inquiries can be directed to the corresponding authors.
